# Partitioning of heat production in growing pigs as a tool to improve the determination of efficiency of energy utilization

**DOI:** 10.3389/fphys.2013.00146

**Published:** 2013-06-19

**Authors:** Etienne Labussière, Serge Dubois, Jaap van Milgen, Jean Noblet

**Affiliations:** ^1^INRA - UMR PegaseSaint-Gilles, France; ^2^Agrocampus Ouest - UMR PegaseRennes, France

**Keywords:** male pig, castrated pig, energy expenditure, physical activity, energy requirements

## Abstract

In growing pigs, the feed cost accounts for more than 60% of total production costs. The determination of efficiency of energy utilization through calorimetry measurements is of importance to sustain suitable feeding practice. The objective of this paper is to describe a methodology to correct daily heat production (HP) obtained from measurements in respiration chamber for the difference in energy expenditure related to physical activity between animals. The calculation is based on a preliminary published approach for partitioning HP between HP due to physical activity (AHP), thermic effect of feeding (TEF) and basal metabolic rate (fasting HP; FHP). Measurements with male growing pigs [mean body weight (BW): 115 kg] which were surgically castrated (SC), castrated through immunization against GnRH (IC), or kept as entire male (EM) were used as an example. Animals were fed the same diet *ad-libitum* and were housed individually in two 12-m^3^ open-circuit respiration chambers during 6 days when fed *ad-libitum* and one supplementary day when fasted. Physical activity was recorded through interruption of an infrared beam to detect standing and lying positions and with force transducers that recorded the mechanical force the animal exerted on the floor of the cage. Corrected AHP (AHP_c_), TEF (TEF_c_), and HP (HP_c_) were calculated to standardize the level of AHP between animals, assuming that the ratio between AHP_c_ and ME intake should be constant. Inefficiency of energy utilization (sum of AHP_c_ and TEF_c_) was lower than the inefficiency estimated from the slope of the classical relationship between HP_c_ and ME intake but was associated with higher requirements for maintenance. Results indicate that EM pigs had higher FHP but lower TEF_c_ than IC and SC pigs. These results agree with the higher contents in viscera of EM pigs that stimulate their basal metabolic rate and with the reduced utilization of dietary protein to provide energy for maintenance energy requirements and fat deposition (FD).

## Introduction

In growing pigs, feeding accounts for more than 60% of total production costs. The increased use of crop resources for human consumption or fuel production in a context of constrained land resources promotes feedstuff diversification in pig diets, including the use of increasing amounts of by-products (Martin, 2010). Nevertheless, these new feedstuffs are often poorly documented for their energy values, whereas the technological treatments they undergo, often associated with high contents in dietary fiber, may strongly affect metabolic utilization of energy by the growing pigs. Different feeding systems (from digestible energy to net energy, NE) that take into account different energy losses by the animal can be used to describe dietary energy value (Baldwin, 1995a). Among them, the NE system requires measuring energy expenditure associated with the utilization of these feedstuffs for growth (or heat increment HI). The direct measurement in growing animals of heat production (HP) in respiration chamber offers the opportunity to evaluate variation among animals in line with their genotype, phenotype or environmental conditions. Nevertheless, animals produce heat because of different metabolic processes involved in their maintenance and growth functions. The calculation of HI in growing animals needs the partitioning of total HP between a component due to maintenance and a component due to growth. Differences in energy expenditure due to different levels of physical activity between animals have also to be accounted for. The objectives of the paper are to present the methodology developed in our laboratory to calculate HI, using a mathematical model previously described (van Milgen et al., 1997). Further calculations to standardize HI for difference in physical acitivty between animals are proposed. An experiment in which the energy expenditure was measured in entire male (EM) and castrated pigs is used as an example.

## Materials and methods

The experiment complied with French laws on animal experimentation and was conducted under the direction of Jean Noblet and Jaap van Milgen, who are both authorized by the French Ministry of Agriculture (n° 4739 and 7704).

### Experimental design

The experiment was designed to determine the effects of castration and castration method on nitrogen and energy metabolism of male growing pigs. The experiment was conducted on six groups of three Pietrain × (Large White × Landrace) male pigs that were either surgically castrated (SC), immunocastrated (IC), or kept as EM. Within each group, pigs originated from the same litter (five groups) or had the same father (one group) to reduce possible bias in their energy metabolism induced by difference in their genotype. Measurements consisted in 6 days when fed for measuring nitrogen and energy balances (difference between intake and losses in feces, urine and as CH_4_ and HP) and 1 day for quantifying fasting HP (FHP) when pigs received no feed. Measurements for IC occurred 5 weeks after the second vaccination when hormonal status of IC pigs was stabilized (Kubale et al., 2013) and measurements for SC and EM pigs occurred simultaneously or 1 week before because only two respiration chambers were available. During measurements, pigs were placed in a metabolic cage allowing quantitative and separate collection of feces and urine and housed individually in a 12-m3 open-circuit respiration chamber, similar as those described by Vermorel et al. (1973). The temperature and relative humidity in the respiration chambers were kept constant at 24°C and 70%, respectively. The pigs were offered a cereal-based diet *ad-libitum* into a trough with a trap door (Table 1). A feed hopper placed above the trough ensured that feed was available during the whole day.

**Table 1:** **Composition of diet**.

**INGREDIENTS (%)**	
Corn	16.00
Wheat	26.20
Barley	25.55
Soybean meal	19.00
Vegetable fat	2.00
Molasses	3.00
Wheat bran	5.00
Bicalcium phosphate	0.50
Calcium carbonate	1.29
Sodium chloride	0.45
L-lysine-HCl	0.33
DL-methionine	0.04
L-threonine	0.03
Vitamins, oligoelements and phytase	0.61
**CHEMICAL COMPOSITION (% OF DM)**	
Crude protein	20.11
Starch	44.66
Crude fat	4.0
Crude fiber	4.0
NDF	17.0
ADF	4.9
ADL	0.9
Gross energy (MJ/kg DM)	18.61

### Measurements and samplings

Pigs were weighed on the morning of the first day of measurements, on the morning of the fasting day and on the morning after the fasting day. The amount of feed offered was recorded daily and feed refusals and spillages were collected at the end of the 6 fed days. Offered feed was sampled daily for each week of measurements. At the end of each week, feces from each pig were weighed, mixed, and sampled. Urine was weighed daily and a daily aliquot was cumulated over the 6 days of the fed period for each pig. Ammonia losses that resulted from the degradation of urinary nitrogen were recovered from the condensed water from the air conditioning system while ammonia losses in outgoing air were determined as described by Noblet et al. (1987).

According to the open-circuit respiration chamber technique, volumes of O_2_ consumption and CO_2_ and CH_4_ production were calculated from ventilation rate of the respiration chamber and from the difference in gas concentrations between outgoing and ingoing air. The O_2_, CO_2_, and CH_4_ concentrations in outgoing air were measured using a paramagnetic differential analyzer (Oxymat 6, Siemens) and two infrared analyzers (Ultramat 6, Siemens), respectively. The ventilation rate was measured with a mass gas meter (Teledyne Brown Engineering). Gas concentrations, ventilation rate and physical characteristics of the gas in the respiration chamber (pressure, temperature, and relative humidity) were measured 60 times per second, averaged over 10-s intervals and recorded for further calculations. Each day, access to the feeder was blocked at 6.00 am and measurements were stopped at 8.00 am for ~15 min to provide care to the animals, refill the feeders, collect feces and urine and calibrate the analyzers with ingoing air as baseline and a gas tube with known gas concentrations as standard. Measurements then restarted and access to feeder was allowed at 9.00 am.

Feeding behavior was recorded continuously using a weighing scale that was placed under the trough. Standing duration was recorded through interruption of an infra-red beam that was placed across the cage at the height of the pig's hip. The mechanical force the pig exerted because of physical activity was recorded using four force sensors (9104A, Kistler) on which the cage was placed. The sensors are transducers that produce an electrical signal proportional to the vertical force the animal exerts on the cage (Quiniou et al., 2001).

### Laboratory analyses

Feed samples and feed refusals were analyzed weekly for dry matter (DM) content. Feed samples were then pooled and analyzed for DM, nitrogen (Dumas method) and energy contents (AOAC, 1990; AFNOR, 1998). One sample of feces per pig was analyzed for DM content and one sample was freeze-dried. Freeze-dried feces samples were ground through a 1 mm grid and analyzed for DM, nitrogen (Dumas method) and energy contents. Nitrogen content of urine was measured on fresh material according to the Dumas method and energy content was measured after freeze-drying approximately 30 mL of urine in polyethylene bags (AFNOR, 1998).

### Calculations

Gas analyzers were calibrated at the beginning and at the end of each day and the drift was considered to be linear. The time lag between respiration chamber and gas analyzers equaled 70 s. Taking into account the effect of respiratory quotient (RQ, CO_2_/O_2_) on difference between inflow and outflow (Ortigues et al., 1994), volumes of O_2_ consumption and CO_2_ and CH_4_ production were calculated for 10-s intervals that were cumulated over the day. To account for the interruption of the measurements in the morning (calibration of analyzers …), these volumes were standardized for 24-h period, assuming proportionality.

Nitrogen balance was calculated as the difference between intake (calculated as the difference between offered feed and feed refusals and spillages) and losses in feces and urine and as ammonia. Protein deposition (PD) was then calculated, assuming that PD equaled 6.25 times nitrogen retention. Retained energy (RE) was calculated as the difference between feed gross energy intake and energy losses in feces and urine and as CH_4_ (39.5 kJ/L of CH_4_) and HP. According to the Brouwer (1965) equation, HP was calculated from volumes of O_2_ consumption, CO_2_ production, and CH_4_ production and nitrogen excreted in urine (including ammonia losses). Fat deposition (FD) was calculated from the energy balance, assuming that energy was retained only as protein (23.6 kJ/g PD) and as fat (39.7 kJ/g FD).

### Mathematical modeling of HP partition

**Figure 1:**
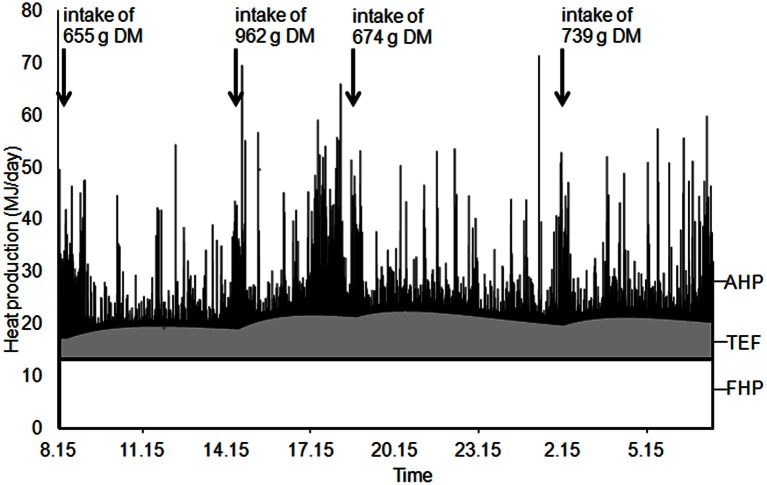
**Example of heat production partitioning between components due to basal metabolic rate (fasting heat production, FHP), physical activity (AHP) and thermic effect of feeding (TEF); IC pig from group 3**.

**Figure 2:**
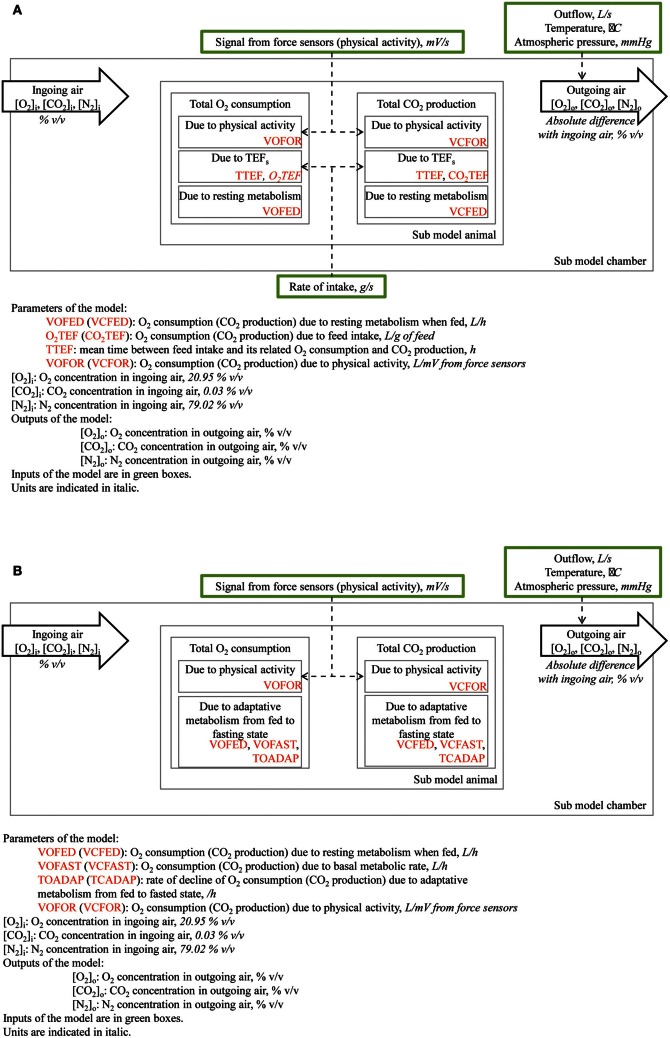
**Description of the mathematical model used to partition total heat production from kinetics of O_2_ consumption and CO_2_ production; (A)** components when animals are in a fed state; **(B)** components when animals are in a fasted state.

Total HP was partitioned between components due to basal metabolism FHP, physical activity (AHP) and thermic effect of feeding (TEF, Figure 1) through analysis of the dynamic patterns of O_2_ and CO_2_ concentrations in the air of the respiration chamber (van Milgen et al., 1997). The model assumes that the instantaneous variations in O_2_ and CO_2_ concentrations are related to O_2_ consumption and CO_2_ production by the pig (sub-model “animal”; Figure 2), in addition to variation induced by ventilation of the respiration chamber and variation of physical characteristics of the gas within the respiration chamber (sub-model “chamber”). A complete description of the mathematical model is given by van Milgen et al. (1997).

#### Mathematical modeling of gas exchanges

The conception of the model was similar for O_2_ consumption and CO_2_ production. During the fed days, the sub-model “animal” considered that instantaneous O_2_ consumption or CO_2_ production (in standard conditions of temperature and pressure: 0°C and 1 atm) equaled the sum of O_2_ consumption or CO_2_ production due to physical activity and short-term TEF (TEF_s_), in addition to constant O_2_ consumption or CO_2_ production associated with resting metabolism (VOFED and VCFED, respectively; Figure 2). It was hypothesized that O_2_ consumption or CO_2_ production due to physical activity was proportional to the electrical signal from force sensors with different parameters for O_2_ and CO_2_ (VOFOR and VCFOR, respectively). The O_2_ consumption or CO_2_ production due to TEF_s_ followed a gamma distribution. The latter was modeled as the output of a two-compartment system, which was filled in the first compartment by feed intake (recorded by the weighing scale placed under the trough) and parameterized by the volume of O_2_ consumed or CO_2_ produced per g of feed intake (O2TEF and CO2TEF, respectively) and by mean time between feed intake and its related O_2_ consumption or CO_2_ production (TTEF). Mathematically, the content of each compartment was modeled from its first-order derivative with respect to time and fractional emptying rates were assumed to be identical for both compartments (2/TTEF; van Milgen et al., 1997). In addition to these well-identified contributors to O_2_ consumption and CO_2_ production, early experiments indicated that modest and time-limited variations in O_2_ and CO_2_ concentrations in the respiration chamber can occur irrespective of feed intake or physical activity (van Milgen and Noblet, 2000). Although the contribution of these phenomena to the total volumes of O_2_ consumption or CO_2_ production is small (<0.5%), they can affect the estimates of parameters of the model when not accounted for. These events were manually included in the model to ensure proper parameter estimation as instantaneous O_2_ consumption and CO_2_ production. During the fasting day, there is no feed intake. However, O_2_ consumption or CO_2_ production during resting (when the contribution of physical activity was removed) are lower during fasting than when fed. The decline in O_2_ consumption or CO_2_ production was described as a first-order decline between O_2_ consumption or CO_2_ production at a fed state (VOFED and VCFED, respectively) and O_2_ consumption or CO_2_ production during fasting (VOFAST and VCFAST, respectively). It was hypothesized that the rate of decline (TOADAP and TCADAP, respectively) may be different for O_2_ and CO_2_. Finally, the sub-model “animal” allowed calculating O_2_ consumption and CO_2_ production using feed intake and signals from the force sensors as inputs and seven parameters for the fed days and eight parameters for the fasting day.

The sub-model “chamber” described the variation in physical characteristics of the gas and considered that the air in the respiration chamber was composed of O_2_, CO_2_, and N_2_. Because only the flow of outgoing air was measured, the inflow was calculated as the flow of air required to fill the physical volume of the respiration chamber when O_2_ consumption, CO_2_ production and outflow were considered; the physical volume of the respiration chamber was calculated in standard conditions of temperature and pressure (0°C, 1 atm). The concentration of each gas in the respiration chamber was then calculated from its volume divided by the sum of volumes of O_2_, CO_2_, and N_2_.

Equations of the model were written in Fortran and compiled in a dynamic linked library that was loaded in R (R Development Core Team, 2010). Package deSolve (Soetaert et al., 2010) was used to solve the ordinary differential equations with an integration step-size of 10 s, after smoothing the data from force sensors, temperature, pressure and outflow to ensure their continuity. Parameters of the model were estimated for each day according to a three-step procedure: parameters directly related to O_2_ consumption or CO_2_ production were first estimated separately and then together to minimize the sum of squared differences between predicted O_2_ or CO_2_ concentration and measured O_2_ or CO_2_ concentrations (Nelder and Mead, 1965).

#### Calculation of HP components

Energy expenditure due to fasting metabolism, physical activity and TEF_s_ were calculated from the respective volumes of O_2_ consumption and CO_2_ production according to the Brouwer (1965) equation. The difference between resting HP when fed (i.e., total HP minus AHP and TEF_s_) and FHP was attributed to long-term TEF (TEF_l_) and total TEF was calculated as the sum of short- and long-term components.

#### Standardization of HP for differences in physical activity

Preliminary analysis on data indicated that correlation between AHP and ME intake was significant (*r* = 0.56, *P* < 0.05; Table 2). To standardize HP between animals for difference in their physical activity, it was assumed that a fixed amount of metabolizable energy (ME) intake should be dissipated as corrected AHP (AHP_c_). The proportion of ME intake that was dissipated as HP due to physical activity equaled the mean value of AHP/ME (8.6%; see results). When AHP was higher than AHP_c_, the difference between AHP and AHP_c_ resulted in a positive variation of ME available for other metabolic pathways, which was dissipated as TEF or retained as fat. The amount which was dissipated as supplementary TEF was calculated as: (AHP—AHP_c_) × TEF / (ME—FHP—AHP) and was added to TEF to calculate a corrected TEF (TEF_c_). The difference between AHP and AHP_c_ which was not dissipated as supplementary TEF was added to RE to calculate corrected RE (RE_c_) and FD (FD_c_). When AHP was lower than AHP_c_, the standardization followed the same calculation routine but resulted in lower TEF_c_, RE_c_ and FD_c_ than TEF, RE, and FD, respectively. Assuming that FHP is representative of the basal metabolic rate of *ad-libitum* fed animals (Baker et al., 1991), HI was calculated as the sum of AHP_c_ and TEF_c_. The efficiency of utilizing ME for maintenance and growth (k_mg_, %) was calculated as (1-HI/ME) × 100. Maintenance ME requirements (ME_m_) were calculated as FHP × 100/k_mg_ (Labussière et al., 2011). All energy traits were expressed relative to metabolic body size, which was calculated as body weight (BW) raised to the power 0.60 (Noblet et al., 1999).

**Table 2:** **Pearson correlation coefficients between time spent standing (h/d), mean voltage measured from force sensors (mV/d), ME intake (kJ/kg BW^0.60^ per day) and physical activity heat production (AHP; kJ/kg BW^0.60^ per day) and their ratio (AHP/ME; %)**.

	**Mean voltage from force sensors**	**ME intake**	**AHP**	**AHP/ME**
Time spent standing	0.12	0.19	0.03	−0.09
Mean voltage from force sensors		0.65^**^	0.85^**^	0.52^*^
ME intake			0.56^*^	−0.09
AHP				0.77^**^

* P < 0.05;

** *P < 0.01*.

The NE intake was calculated as the difference between ME intake and HI. The energy values of the diet (ME and NE contents) were calculated as the ratio between ME or NE intake (MJ/day) and feed intake.

### Statistical analyses

One FHP value was missing for a SC pig in group 2 and it was calculated (kJ/kg BW^0.60^ per day) as the average of the values obtained for the SC in the five other groups. The data (*n* = 18) were analyzed for the effects of sex (EM, SC, IC) and group using the PROC GLM of SAS (SAS, 2004). Only the *P*-values for the effect of sex will be described in details. Pearson correlation coefficients between time spent standing, mean voltage measured from force sensors, ME intake, AHP and AHP/ME ratio were calculated (PROC CORR; SAS, 2004). The linear relationship between AHP (% of ME intake) and the mean voltage from force sensors (mV/day) was tested and the difference of the slope from zero was tested through a *T*-test (PROC GLM; SAS, 2004). The linear relationship between corrected HP_c_ (HP_c_) (kJ/kg BW^0.60^ per day) and ME intake (kJ/kg BW^0.60^ per day) was tested for the effect of sex on intercept and slope of the relationship (PROC GLM; SAS, 2004).

## Results

### Methodological aspects

**Figure 3:**
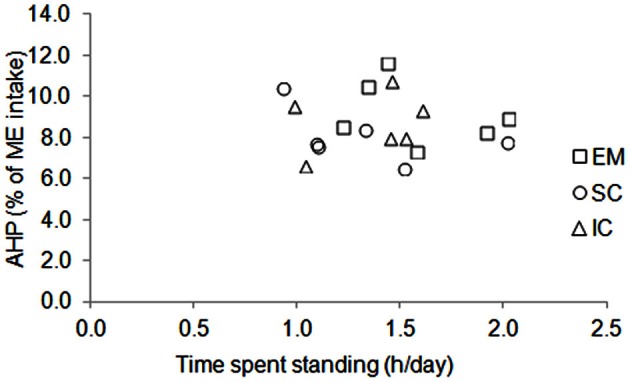
**Individual variations of time spent standing and energy expenditure due to physical activity (AHP, % of ME intake) in entire male (EM), surgically castrated (SC), and immune-castrated (IC) pigs**.

**Figure 4:**
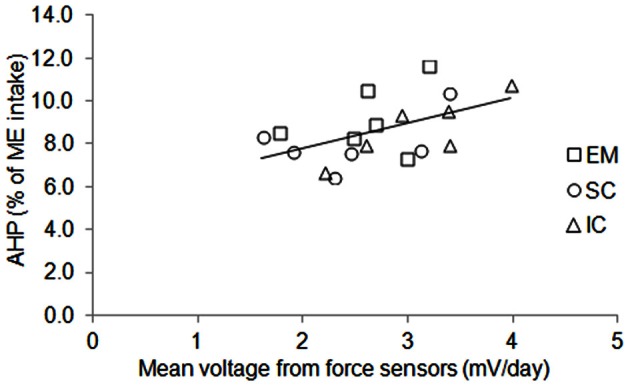
**Individual variations of mean voltage measured from force sensors and energy expenditure due to physical activity (AHP, % of ME intake) in entire male (EM), surgically castrated (SC) and immune-castrated (IC) pigs**. Solid line: linear relationship between AHP (% of ME intake) and cumulative voltage from force sensors (mV/day); the slope equaled 1.2% of ME per mV and differed significantly from zero (*P* = 0.03).

The BW of the pigs did not differ between sexes and averaged 115 kg during measurements (Table 3). Voluntary ME intake varied significantly between 2396 kJ/kg BW^0.60^ per day for EM pigs to 2864 kJ/kg BW^0.60^ per day for IC pigs. There was no effect of sex on time spent standing that averaged 1.4 h/day but individual values varied from 0.9 to 2.0 h/day (Figure 3). The force the animals exerted on the floor (mean voltage measured from force sensors) varied from 1.6 to 4.0 mV/day (Figure 4) and it was significantly correlated with ME intake, AHP and AHP/ME intake (Table 2). The AHP did not differ significantly between sexes (Table 3) but it was significantly correlated with ME intake (Table 2). When expressed as a percentage of ME intake, AHP was not affected by sex and averaged 8.6% (Table 3). Additionally, it was significantly correlated with mean voltage from force sensors (Table 2).

**Table 3:** **Effect of castration and castration method on energy balance, efficiency of utilizing ME for maintenance and growth and maintenance energy requirements in male growing pigs (results are LS-means; *n* = 18)**.

	**Sex**				
	**EM**	**SC**	**IC**	**Rsd**	***P*-value**
BW (kg)	114.0	111.0	120.1	7.5	0.15
Time spent standing (h/day)	1.6	1.3	1.3	0.4	0.42
ME intake (kJ/kg BW^0.60^ per day)	2396^b^	2632^a^^,^^b^	2864^a^	208	<0.01
**ENERGY EXPENDITURE (kJ/kg BW^0.60^ PER DAY)**					
FHP	856^a^	735^b^	783^b^	36	<0.01
AHP	218	212	250	38	0.22
AHP_c_	207^b^	227^a^^,^^b^	247^a^	18	<0.01
TEF_c_	315^b^	464^a^	484^a^	65	<0.01
HI	522^b^	692^a^	732^a^	81	<0.01
HP_c_	1376	1416	1519	91	0.06
RE_c_ (kJ/kg BW^0.60^ per day)	1020^b^	1216^a^	1346^a^	125	<0.01
**ENERGY EXPENDITURE (% of ME INTAKE)**					
AHP	9.2	8.0	8.7	1.5	0.45
AHP_c_	8.6	8.6	8.6	−	
TEF_c_	13.0^b^	17.5^a^	16.9^a^	1.6	<0.01
HI_c_	21.6^a^	26.2^b^	25.6^b^	1.6	<0.01
ME_m_ (kJ/kg BW^0.60^ per day)	1091^a^	997^b^	1054^a^^,^^b^	42	0.02
Respiratory quotient	1.08^b^	1.14^a^	1.15^a^	0.03	<0.01
**DIETARY ENERGY VALUE (MJ/kg DM)**					
ME	15.41	15.37	15.13	0.19	0.06
NE	12.02^a^	11.42^b^	11.25^b^	0.35	<0.01

*EM, entire male pigs; SC, surgically castrated pig; IC, immuno-castrated pig; Rsd, residual standard deviation; BW, body weight; ME, metabolizable energy; FHP, fasting heat production; AHP, physical activity related heat production; TEF_c_, thermic effect of feeding corrected for inter-individual differences in energy expenditure due to physical activity; HI, heat increment; HP_c_, total heat production corrected for inter-individual differences in energy expenditure due to physical activity; RE_c_, retained energy corrected for inter-individual differences in energy expenditure due to physical activity; ME_m_, maintenance metabolizable energy requirements; NE, net energy; Measurements started 5 weeks after the second vaccination for IC pigs. Only 5 data for SC pigs were available for calculating FHP*.

a, b *Within the same row; LS-means with different superscripts differ (P < 0.05)*.

### Energy balance

Total HP_c_ tended to vary according to the same pattern as ME intake from 1376 to 1519 kJ/kg BW^0.60^ per day (*P* = 0.06; Table 3). The relationship between HP_c_ and ME intake did not differ significantly between sexes; the intercept equaled 554 kJ/kg BW^0.60^ per day and the slope equaled 34%. Among HP_c_ components, FHP was significantly higher for EM pigs (856 vs. 761 kJ/kg BW^0.60^ per day on average for castrated pigs) whereas TEF_c_ was significantly lower for EM pigs (315 vs. 474 kJ/kg BW^0.60^ per day or 13.0 vs. 17.2% of ME intake on average for castrated pigs). When HP due to physical activity was corrected for the differences between animals, HI was significantly lower in EM pigs than in castrated pigs (522 vs. 712 kJ/kg BW^0.60^ per day, on average). Variations in ME intake and energy expenditure resulted in lower RE_c_ in EM than in castrated pigs (1020 vs. 2562 kJ/kg BW^0.60^ per day, on average). Additionally, inefficiency of utilizing ME for maintenance and growth (i.e., HI_c_ expressed as % of ME intake) was significantly lower in EM than in castrated pigs (21.6 vs. 25.9%, on average for castrated pigs). Maintenance ME requirements varied among pigs and ranged from 997 for SC pigs to 1091 kJ/kg BW^0.60^ per day for EM pigs. The RQ was significantly lower in EM pigs than in castrated pigs, irrespective of castration method (1.08 vs. 1.15). Dietary ME content tended to vary between 15.13 in IC pigs to 15.41 MJ/kg DM in EM pigs. The NE content of the diet was significantly higher for EM pigs (12.02 vs. 11.34 MJ/kg DM on average for castrated pigs).

### Nutrient deposition

The BW gain was calculated from BW measured at the beginning and at the end of the 6 days of balance measurement; it did not differ between sexes and averaged 1273 g/day (Table 4). From balance measurements, PD was significantly lower for SC pigs (196 vs. 254 g/day on average for EM and IC pigs) whereas FD_c_ was significantly lower for EM pigs (288 vs. 429 g/day on average for SC and IC pigs). Accordingly, the FD_c_ content of BW gain was lower for EM pigs whereas the PD content of BW gain did not differ significantly between sexes and averaged 234 g/day.

**Table 4:** **Effect of castration and castration method on BW gain and its composition in male growing pigs (results are LS-means; *n* = 18)**.

	**Sex**				
	**EM**	**SC**	**IC**	**Rsd**	***P*-value**
BW gain (kg/day)	1370	1133	1317	288	0.37
**NUTRIENT DEPOSITION (g/day)**					
PD	261^a^	196^b^	246^a^	28	<0.01
FD_c_	288^b^	403^a^	454^a^	51	<0.01
**BODY GAIN COMPOSITION (g/kg of BW GAIN)**					
PD	261	196	246	43	0.53
FD_c_	284^b^	407^a^	453^a^	41	<0.01

*EM, entire male pigs; SC, surgically castrated pig; IC, immune-castrated pig; Rsd, residual standard deviation; BW, body weight; PD, protein deposition; FD_c_, fat deposition corrected for inter-individual differences in energy expenditure due to physical activity; Measurements started 5 weeks after the second vaccination for IC pigs*.

a, b *Within the same row; LS-means with different superscripts differ (P < 0.05)*.

## Discussion

### Methodological aspects for measuring net energy value of a diet

The evaluation of the energy value of feedstuffs and feeds requires estimating the efficiency of energy utilization of nutrient utilization by animals. In growing animals, theoretical calculations involve the artificial distinction between energy use for maintenance and for growth and require several assumptions regarding metabolic pathways and composition of BW gain (protein and lipid deposition, protein and lipid turnover, fatty acid composition of *de novo* lipid synthesis). Additionally, these calculations do not account for the energy costs associated with ingestion and digestion of feed. Alternatively, calorimetry measurements in living animals allow estimating an overall efficiency of utilizing dietary energy for maintenance and growth and they include the associated energy costs. In this paper, efficiency was calculated from the inefficiency due to TEF and AHP. Nevertheless, it could be biased by differences in physical activity among animals (i.e., social confinement, reduced physical activity because of contention).

Several methods have been used in the past to quantify physical activity and to link physical activity to energy expenditure. In pigs, these methods have been based on regression analyses between HP and time budget that was determined using infrared barriers (e.g., Noblet et al., 1993) or video recordings (e.g., Rijnen et al., 2003) but these methods do not allow quantifying the level of physical activity (i.e., the mechanical force and the associated efficiency the animal develops because of its physical activity). In this way, results from our experiment indicate that the time the animals spent standing has little effect on AHP (Figure 3). Indeed, the time the pig was standing was measured through an infra-red barrier, which was placed across the cage at the height of the pig's hip. Consequently, standing also included other activities like sitting, rubbing, walking (only to small extent because of the cage), or digging. The quantification of physical activity requires measuring traits which are thought to be proportional to the mechanical force which is exerted by the animal. Indeed, ultrasonic burglars were used in pigs (e.g., Schrama et al., 1998) and more recently, accelerometers have been proposed to measure physical activity in rodents and humans. Nevertheless, these measurements may be subject to errors in estimating accurately physical activity of large animals because measured values can be specific to a given physical activity. The consequence is that measured values can be less representative of the physical activity of the whole body, depending on the position of the ultrasonographic burglar devices relative to the body of the animal, or the position of the accelerometer on the body of the animal. In our experiments and in others (e.g., Even et al., 1991), the cage where the animals were housed was located on force transducers that are sensors that produce an electrical signal proportional to the force the animal exerts on the floor. The partitioning of total HP to determine what is due to physical activity then requires estimating the amount of energy expenditure per unit of electrical signal from force sensors and involves parameter optimization through mathematical modeling. Using the signals from force transducers, the latter has been performed through Kalman filtering (Kalman, 1960; Even et al., 1991) or Nelder–Mead minimization (Nelder and Mead, 1965; van Milgen et al., 1997). In our approach, parameter optimization includes also the determination of energy cost associated with TEF and resting metabolism. In this paper, the determination coefficient of the variations in gas concentrations by the mathematical model averaged 92% over the 126 days that were modeled (18 pigs with 7 days each). Nevertheless, the model considers that each unit of electrical signal from force transducers corresponds to a fixed volume of consumed O_2_ and produced CO_2_ and does not account for the metabolic difference in muscles involved in physical activity between standing and lying.

### Efficiency of utilizing energy in growing pigs

Growing animals produce heat because of their maintenance and growing metabolism. Classically, the slope of the relationship between HP_c_ and ME intake (34% in our experiment) was considered as an estimate of the inefficiency of utilizing dietary energy but this approach has been questioned because of the adaptation of animal to feeding level (de Lange et al., 2006; Labussière et al., 2011). In the modelling approach for partitioning HP, AHP and TEF are indicative for the inefficiency in utilizing dietary energy whereas FHP is indicative of the basal metabolic rate of animals (Labussière et al., 2011). This inefficiency varied from 22% in EMs to 26% in castrated pigs which agrees with previous results (Labussière et al., 2011). These values were also lower than those estimated from the classical regression between HP_c_ and ME intake but they were associated with higher values of maintenance energy requirements (Labussière et al., 2011).

Irrespective of castration method, AHP accounted for 8.6% of ME intake, which agrees with previous observations in growing pigs fed close to *ad-libitum* (from 7.6 to 11.6% of ME intake; Schrama et al., 1998; Le Bellego et al., 2001; Quiniou et al., 2001; van den Borne et al., 2007; Labussière et al., 2011; Renaudeau et al., 2013) but values were highly variable between animals (Figure 3). To account for the possible bias induced by the variation in AHP between animals, a calculation routine was used to standardize AHP between animals, which resulted in variations in TEF_c_ and RE_c_. In our experiment, TEF_c_ was higher in SC and IC pigs than in EM (Table 3). Values for SC or IC pigs agree with previous results in SC pigs which received a similar diet (16.8% of ME intake; Barea et al., 2010). Data for TEF_c_ in EM pigs are scarce but the differences in TEF_c_ between EMs and castrated pigs agree with the differences in metabolism of nutrients due to lower feed intake, higher PD and lower lipid deposition that result in a lower RQ in EM pigs. Indeed, theoretical calculations for energy efficiencies for lipid deposition are always lower when the energy is provided by proteins rather than by carbohydrates or lipids (Armstrong, 1969). Calculations using diet composition and the difference between digested N and N deposited in PD (Table 4) indicate that dietary protein contributed to 13% in EM and 18% in SC of the energy used for maintenance and lipid deposition, which agrees with the lower TEF_c_ in EM pigs. Consequently, dietary NE content, which is thought to be representative of the true energy value of the diet, depended on the sexual type of the animal and it was higher in EM pigs (Table 3).

### Maintenance energy metabolism in growing pigs

During the fasting day, the mathematical modeling of HP was considered to occur according a first-order decrease in energy metabolism between fed and fasted states. The FHP was calculated as the asymptotic value of resting HP (van Milgen et al., 1997). These values of FHP exclude the energy expenditure due to physical activity and the values for SC pigs agree with values previously estimated using a similar methodology (711 to 846 kJ/kg BW^0.60^ per day; Le Bellego et al., 2001; Le Goff et al., 2002; Lovatto et al., 2006; Barea et al., 2010; Labussière et al., 2011). According to previous results with growing pigs (van Milgen et al., 1998), FHP of EM pigs was higher than that of castrated pigs (Table 3). This result agrees with the greater mass of viscera in EM than in SC (Quiniou and Noblet, 1995), which influences FHP (Koong et al., 1982, 1985; Pekas and Wray, 1991) because of the greater energy requirements of the portal-drained viscera (Johnson et al., 1990; Ortigues et al., 1995). Estimating FHP allows determining ME_m_ in growing animals as the ratio between FHP and k_mg_ (Labussière et al., 2009) without involving the classical regression analyses between RE and ME intake (Kielanowski, 1965; Baldwin, 1995b). The classical regression analysis has been criticized because of the adaptation of the animal to feeding level (de Lange et al., 2006; Labussière et al., 2011). In our experiment, ME_m_ was higher in EM than in SC pigs, which disagrees with previous results (Noblet et al., 1999). Nevertheless, the latter values were calculated from the classical regression methods and were obtained with pigs at lower BW (i.e., younger) than those in the present study. The difference in energy metabolism between entire and castrated males may be less pronounced because of less advanced sexual maturity.

In conclusion, mathematical modeling of daily dynamics of HP and accounting for the variation in physical activity among animals allows calculating the energy expenditure due to physical activity and TEF, in addition to the HP due to basal metabolic rate. In growing animals, the energy utilization of the diet depends on metabolic pathways involved in maintenance and lipid deposition, according to the nutrients that are used. Consequently, the dietary NE content depends on the sexual type of growing animals.

### Conflict of interest statement

The authors declare that the research was conducted in the absence of any commercial or financial relationships that could be construed as a potential conflict of interest.
